# Rare DCM associated variants in pre-miR-208a disrupt miRNA maturation and function

**DOI:** 10.1093/hmg/ddaf069

**Published:** 2025-05-06

**Authors:** Yolan J Reckman, Jan Haas, Ingeborg van der Made, Simon G Williams, Iria Gomez Diaz, Mohammed Akhtar, Jens Mogensen, Torsten B Rasmussen, Eric Villard, Philippe Charron, Perry Elliott, Bernard D Keavney, Lorenzo Monserrat, Yigal M Pinto, Benjamin Meder, Anke J Tijsen

**Affiliations:** Amsterdam UMC, University of Amsterdam, Experimental Cardiology, Amsterdam Cardiovascular Sciences, Meibergdreef 9, 1105 AZ Amsterdam, The Netherlands; Department of Internal Medicine III, Medical Faculty, Heidelberg University, Im Neuenheimer Feld 672, 69120 Heidelberg , Germany; Amsterdam UMC, University of Amsterdam, Experimental Cardiology, Amsterdam Cardiovascular Sciences, Meibergdreef 9, 1105 AZ Amsterdam, The Netherlands; Division of Cardiovascular Sciences, University of Manchester, Oxford Rd, Manchester M13 9PL, United Kingdom; Scientific Department, Health in Code S.L., Av. de Arteixo 43, 15008 A Coruña, Spain; Inherited Cardiac Diseases Unit, The Heart Hospital, University College London, Gower St, London WC1E 6BT, United Kingdom; Department of Cardiology, Odense University Hospital, J. B. Winsløws Vej 4, 5000 Odense, Denmark; Department of Cardiology, Aarhus Universitetshospital, Palle Juul-Jensens Boulevard 69, 8200 Aarhus, Denmark; AP-HP, Department of Cardiology & Department of Genetics, Sorbonne University, INSERM UMRS-1166, ICAN Institute, Pitié-Salpêtrière Hospital, 47-83 Bd de l'Hôpital, 75013 Paris, France; AP-HP, Department of Cardiology & Department of Genetics, Sorbonne University, INSERM UMRS-1166, ICAN Institute, Pitié-Salpêtrière Hospital, 47-83 Bd de l'Hôpital, 75013 Paris, France; Member of the European Reference Network for rare, low prevalence and complex diseases of the heart: ERN GUARD-Heart; Inherited Cardiac Diseases Unit, The Heart Hospital, University College London, Gower St, London WC1E 6BT, United Kingdom; Division of Cardiovascular Sciences, University of Manchester, Oxford Rd, Manchester M13 9PL, United Kingdom; Medical Department, Dilemma Solutions SL, Rúa Antonio Insua Rivas, 56, 15008 A Coruña,Spain; Manchester NIHR Biomedical Research Centre, Manchester University NHS Foundation Trust, Oxford Rd, Manchester M13 9WL, United Kingdom; Amsterdam UMC, University of Amsterdam, Experimental Cardiology, Amsterdam Cardiovascular Sciences, Meibergdreef 9, 1105 AZ Amsterdam, The Netherlands; Member of the European Reference Network for rare, low prevalence and complex diseases of the heart: ERN GUARD-Heart; Department of Internal Medicine III, Medical Faculty, Heidelberg University, Im Neuenheimer Feld 672, 69120 Heidelberg , Germany; Amsterdam UMC, University of Amsterdam, Experimental Cardiology, Amsterdam Cardiovascular Sciences, Meibergdreef 9, 1105 AZ Amsterdam, The Netherlands

**Keywords:** microRNA, Dilated cardiomyopathy, Rare functional variant, miR-208a

## Abstract

Dilated cardiomyopathy (DCM) is a major cause of heart failure (HF) defined by ventricular dilatation and systolic dysfunction. Although microRNAs (miRNAs) are known to affect HF development, little is known about the contribution of genetic variants in miRNAs or their precursors to the susceptibility or pathogenesis of DCM. We screened 1640 DCM cases for variants in cardiac miR-208a and miR-208b and their precursors. We identified four variants in the miR-208a pre-miRNA, which are present at very low frequencies in the general population. Two of these variants (+42G > T and +68G > T) alter a highly conserved nucleotide and the predicted pre-miRNA secondary structure. Both variants result in reduced mature miR-208a levels in overexpression experiments. The variant +42G > T also increased pre-miR-208a levels in these experiments, which indicates a maturation deficiency. Co-transfection of the overexpression constructs with a luciferase construct containing six miRNA binding sites revealed that both variants also impair repression of luciferase expression by miR-208a, indicative of also a loss of miR208a function. Together this indicates that these DCM-associated variants impair formation of mature miR208a. Combined with the role of miR-208a in cardiac contractility this suggests that variants +42G > T and +68G > T in pre-miR-208a may contribute to the DCM phenotype observed in these patients.

## Introduction

Dilated cardiomyopathy (DCM) is defined by ventricular dilatation and systolic dysfunction in the absence of abnormal loading conditions or coronary artery disease [1]. It is one of the major causes of heart failure (HF) and the leading cause of heart transplantation. Familial forms of DCM account for 20%–35% of the cases [2, 3] and at least 50 genes have now been associated with DCM in human and in model systems [1]. However, the genetic make up for DCM is heterogeneous and only in 13%–16% of cases a (likely) pathogenic variant is identified [4, 5]. Furthermore, the highly variable genotype–phenotype relation in DCM and scarcity of large pedigrees suggests that DCM is an oligogenic rather than monogenic disease. Here, we propose that rare functional variants in microRNA (miRNA) encoding genes contribute to the susceptibility for DCM in an oligogenic fashion.

MiRNAs are ~ 22 nucleotide long, single-stranded RNA sequences that regulate gene expression at the post-transcriptional level [6]. They are transcribed from the genome as several thousand nucleotides (nt) long pri-miRNAs, which can contain multiple hairpin structures that each consist of a double stranded RNA (dsRNA) stem flanked by single-stranded (ssRNA) basal segments and an apical loop. Drosha cleaves ~ 11 nt away from the junction between stem and basal segments to produce a single hairpin, the pre-miRNA [7]. The pre-miRNA contains the hairpin structure and dsRNA termini with a typical 5′-phosphate and a 2 nt overhang at the 3’end (8]. Dicer measures ~ 22 nt away from these dsRNA termini to cleave the apical loop of the pre-miRNA and form a miRNA-duplex [8, 9]. Next, Dicer presents one of the strands of this duplex to Argonaute to load it into the RNA-induced silencing complex (RISC), while the other (passenger) strand is degraded. The mature miRNA binds the 3′ untranslated region (3’ UTR) of its target mRNA, to guide the RISC complex to this 3’UTR and inhibit translation. One individual miRNA sequence can bind multiple 3’UTRs and thus regulate multiple genes, therefore, they efficiently regulate almost every cellular process [6].

MiRNA encoding sequences, in particular the pre-miRNA, show high conservation between human individuals [10] and across species [6], which indicates that variants in (pre-)miRNAs are not well tolerated and potentially deleterious. This is supported by studies where germline point mutations or larger genomic deletions affecting miRNAs have been identified as underlying cause for disease [11–14]. Furthermore, also common variants, i.e. single nucleotide polymorphisms (SNP), in miRNAs are associated with disease, including different cardiovascular phenotypes [15, 16]. Variants in miRNA encoding sequences can affect miRNA expression and biogenesis or miRNA function via several mechanisms: 1) Variants in the promotor or enhancer region can affect transcription of the miRNA precursors; 2) Variants in miRNA precursors can alter miRNA maturation; 3) Variants in the mature miRNA can alter binding to 3’UTRs and thereby the genes regulated by this miRNA [16].

In the heart, miRNAs play a crucial role as demonstrated by cardiomyocyte specific Dicer knockout mice, which developed severe HF and arrhythmias leading to sudden cardiac death [17, 18]. Additionally, numerous reports have described both cardiomyocyte and non-cardiomyocyte miRNAs to regulate various stages of HF development [19, 20]. A distinct family of cardiac miRNAs, the so-called myomiRs are encoded by introns of the different myosin heavy chain (MHC) genes, where miR-208a is located in *MYH6*, miR-208b in *MYH7* and miR-499 in *MYH7b*. Interestingly, expression of these myomiRs and their host genes is controlled by one of them, miR208a, which also regulates the ratio between the fast twitch α-myosin heavy chain isoform (αMHC) versus the slow twitch β-isoforms (βMHC and MYH7b) [21, 22]. This α/βMHC ratio is decreased in pressure overload and cardiac stress and determines cardiac contractility, where small changes within the individual sarcomere constitution have profound effects on overall cardiac function [23–26]. Through targets THRAP1, Myostatin, GATA4, HOP and Cx40, miR-208a upregulation promotes hypertrophy, fibrosis and arrhythmias [21, 22, 27].

In this study, we analysed miR-208a and miR-208b and their precursors in 1640 DCM cases previously included in three sequencing studies. We identified four heterozygous variants in pre-miR-208a of which two (+42G > T and + 68G > T) alter a highly conserved nucleotide and the predicted pre-miRNA secondary hairpin structure. We show that these variants impair miR-208a maturation and its capacity for translational inhibition.

## Results

### Multiple variants in miR-208a are enriched in DCM cases compared to the general population

A total of 1640 DCM cases from 3 different cohorts: 1) INHERITANCE 634 cases; 2) 100.000 Genomes Project (100KGP) 538 cases; 3) Health in Code 468 cases, were analysed for variants in miR-208a and miR-208b. All cases were clinically classified as DCM, although as further indicated in the methods section, the exact definition differs per cohort. For all cohorts, only a limited number of clinical characteristics were available (Table 1). Overall, 61.3% of cases were male. The mean age at diagnosis was 32.9 years in INHERITANCE and 35.0 years in 100KGP. Dyspnea was reported in 57.8% of cases in INHERITANCE and 37.0% in 100KGP. Additionally, a positive family history of DCM was observed in 41.6% of cases in INHERITANCE and 69.1% in 100KGP. Left ventricular dimensions (LVEDD) and systolic function (LVEF) were only available for INHERITANCE, which were 64.6 mm and 30.3%, respectively.

**Table 1:** Case characteristics for the INHERITANCE, 100.000 genomes project and health in code cohort.

**Characteristics**	**Total**	**INHERITANCE**	**100.000 Genomes Project**	**Health in Code**
**Number of cases**	1640	634	538	468
**Gender, men (%)**	61.3	63.3	51.3	70.1
**Age of diagnosis (years)**	NA	32.9	35.0^a^	NA
**LVEF (%)**	NA	30.3	NA	NA
**LVEDD (mm)**	NA	64.6	NA	NA
**Dyspnea (%)** **NYHA I** **NYHA II** **NYHA III** **NYHA IV**	NA	57.8^d^ 22.7 25.7 25.9 6.2	37.0^b^	NA
**Positive family history for DCM (%)**	NA	41.6	69.1^c^	NA

Please note that some variables are not available for all cases. NA: not available.

^a^available for 470 cases.

^b^available for 289 cases.

^c^available for 349 cases.

^d^available for 510 cases.

**Figure 1:**
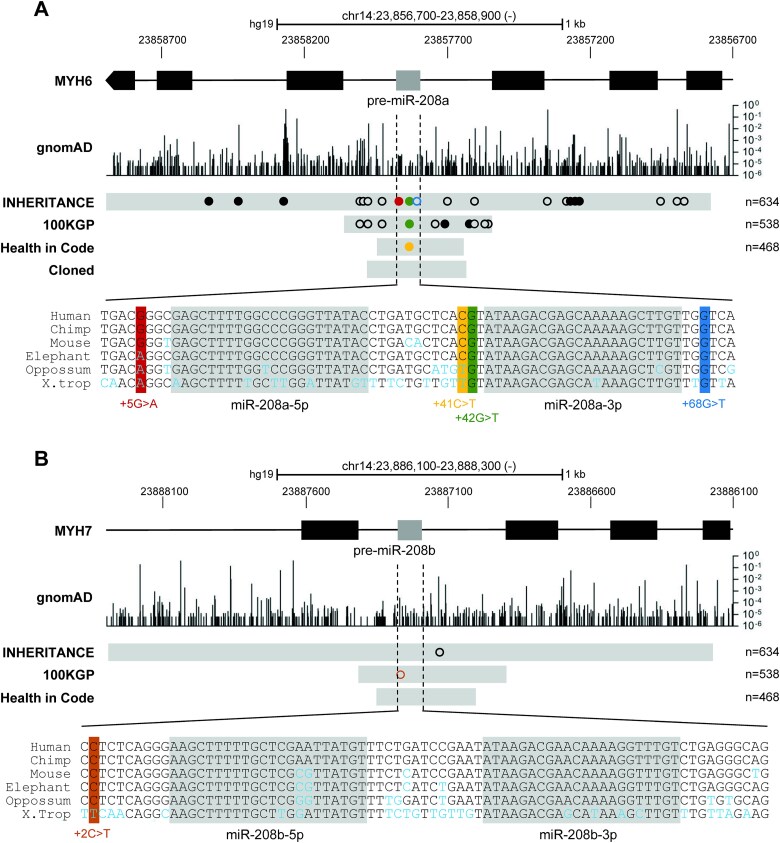
Variants in miR-208a and miR-208b detected in 3 DCM cohorts. Identified variants in the miR-208a (A) and miR-208b locus (B). Top: Genomic position on chromosome 14 (hg19), with miR-208a located in intron 29 of *MYH6* and miR-208a in intron 30 of *MYH7*. Middle: GnomAD traces indicate minor allele frequencies of variants identified in gnomAD on a log(10) scale. INHERITANCE, 100KGP, and health in code indicates per cohort the analysed area in grey, with the identified variants in each cohort indicated by circles (filled: Enriched in respective DCM cohort compared to gnomAD; open: Not enriched). Cloned indicates the cloned miR-208a sequence in grey. Bottom: Sequence conservation of the pre-miRNA, where mature miRNA sequences are highlighted in grey. Identified pre-miRNA variants are highlighted in colour. Variant positions are counted from the first nucleotide of the pre-miRNA sequence.

To study our hypothesis that variants in cardiac-specific miRNAs or their precursors are associated with DCM, we selected the cardiac-specific miRNAs, miR-208a (ENST00000362287) and miR-208b (ENST00000401172) for further analysis within this DCM cohort. We selected those microRNAs because they have previously been associated with cardiac homeostasis [21, 22, 27]. Therefore, sequencing data either obtained by Next Generation Sequencing (INHERITANCE and 100KGP) or Sanger sequencing (Health in Code) of the miR-208a and miR-208b loci were analysed for the occurrence of variants. Within the miR-208a locus we identified 26 variants of which 5 were identified both in INHERITANCE and 100KGP. Compared to the gnomAD population, 9 variants were significantly enriched in one or more DCM cohorts and 2 variants were completely absent in gnomAD. (Fig. 1A, Table S1). For miR-208b we identified only 2 variants, which were both not significantly enriched compared to the gnomAD population (Fig. 1B, Table S1).

### Four very rare heterozygous variants in pre-miR-208a are identified in sporadic DCM cases

Because variants in the pre- or mature miRNA sequence are hypothesized to influence biogenesis and/or target binding, we further focused on variants in these sequences. We identified 4 heterozygous variants in pre-miR-208a: +5G > A (NC_000014.8:23857871C > T), +41C > T (NC_000014.8:23857835G  > A), +42G > T (NC_000014.8:23857834C > T) and + 68G > T (NC_000014.8:23857808C > A), of which +42G > T was identified in 2 unrelated individuals from the INHERITANCE and 100KGP cohorts respectively (Fig. 1A). For the shortened variant notation used in this paper, the position is counted from the first nucleotide of the miRBase annotated pre-miRNA sequence. +5G > A, +41C > T and +42G > T were significantly enriched in their respective DCM cohorts compared to gnomAD. In gnomAD, +5G > A was found in 3 out of 152 186 alleles (OR 40.0; p = 3.1^*^10^−6^), +41C > T in 2 out of 152 158 alleles (OR 81.4; p = 1.2^*^10^−11^) and +42G > T in 1 out of 152 180 alleles (INHERITANCE: OR 120.1; p = 5.2^*^10^−13^/100KGP: OR 141.6; p = 7.3^*^10^−15^). +68G > T was not significantly enriched compared to gnomAD, where it was found in 10 out of 152 204 alleles (OR 12.0; p = 0.0812) (Table S1). No homozygote carriers of the minor allele were reported in gnomAD for these 4 variants. In the pre-miRNA sequence of miR-208b, we identified 1 heterozygous variant (+2C > T) in a DCM case, which was not enriched compared to gnomAD (41 out of 152 238 alleles; OR 4.0; p = 1.0) (Table S1). We did not identify any variant in the mature miRNA sequences of miR-208a or miR-208b.

We further investigated the clinical details of the cases carrying these pre-miR-208a variants. The patient carrying +5G > A is a Danish male who presented at childhood with a severe DCM phenotype for which he underwent urgent heart transplantation. The parents of this patient have not been examined clinically, but appear to be healthy and do not have a history of heart failure. A likely pathogenic variant in TNNT2 was also identified in this patient. Genetic screening of family members was denied by the family. The patient carrying +41C > T is a British male who presented with DCM and severe left ventricular dysfunction around the age of 20. Additional details regarding the phenotype or family members are not available. The patient carrying +42G > T from the INHERITANCE cohort is a British male with impaired left ventricular function combined with conduction disease. Genetic testing was negative for *LMNA* (likely) pathogenic variants. Other genes were not tested. There was no known family history of cardiomyopathy or sudden death and family screening was not performed. An inherited cause for the phenotype was deemed unlikely by the treating physicians. Possibly, the phenotype was related to a previous episode of myocarditis. The patient carrying +42G > T from the 100KGP cohort is a female included as a DCM and conduction defects case around the age of 50. As per inclusion criteria, she suffers from DCM in the absence of disease causing mutations in DCM related genes. Further analysis showed that the mother is carrying the variant of interest, while the father is homozygous for the wildtype allele. Both parents have no known history of DCM. The patient carrying +68G > T is a French female who presented around the age of 70 with heart failure due to DCM with severe left ventricular systolic dysfunction. There was no known family history of DCM and the patient did not have any children. Genetic screening of a panel of DCM associated genes did not identify a (likely) pathogenic variant.

### Variants +42G > T and +68G > T alter a conserved nucleotide, which affects the pre-miR-208a hairpin

To further investigate whether these 4 variants could be biologically important, we determined the level of conservation between species as indication for the selection pressure on this nucleotide and its possible biological importance. This revealed that both +42G > T and +68G > T alter a highly conserved nucleotide, i.e. with a conservation estimate similar to the mean of all nucleotides within pre-miR-208a. On the other hand, +5G > A and +41C > T alter a less conserved nucleotide, i.e. with a conservation estimate lower than the mean of all nucleotides within pre-miR-208a (Fig. 1A and S1).

**Figure 2:**
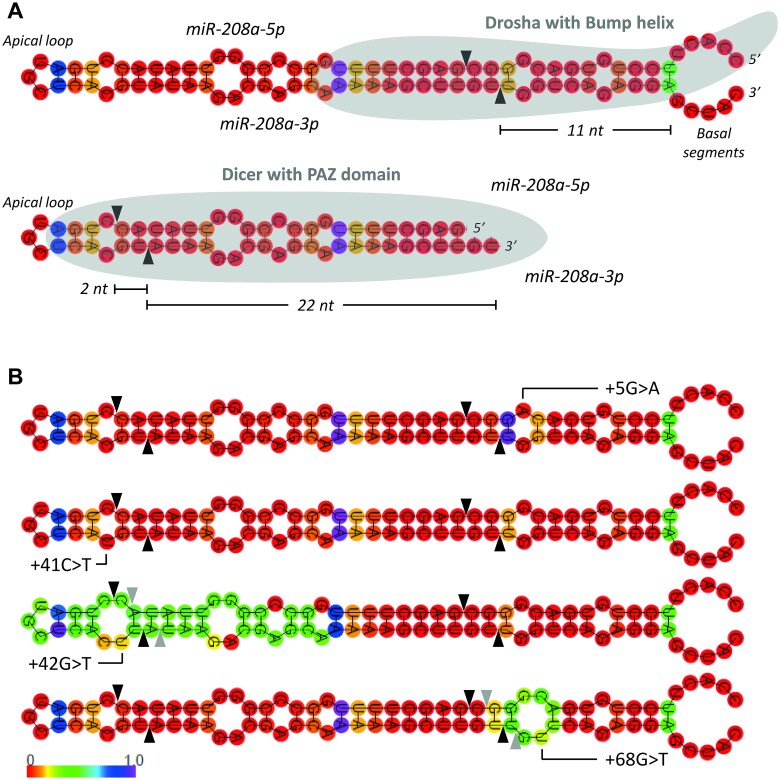
+42G > T and +68G > T affect the pre-miR-208a hairpin structure. (A) Top: miR-208a wildtype hairpin structure and the canonical cleavage site of Drosha ~11 nt away from the junction between stem and basal segments. Bottom: Pre-miR-208a hairpin after Drosha processing and the canonical cleavage site of dicer ~22 nt away from the dsRNA termini. (B) Pre-miR-208a hairpin structures affected by the identified variants, where the position of the specific variant is indicated in the hairpin and the colour of the base indicates the positional entropy as measure for the thermal stability. Canonical Drosha and dicer cleavage sites are indicated by black triangles. Expected non-canonical cleavage sites caused by +42G > T and +68G > T are indicated by grey triangles.

Because the structure of the pre-miRNA hairpin determines whether and where Drosha and Dicer cut this hairpin during miRNA biogenesis, we determined, by using RNAfold [28], whether these variants alter the structure of the wildtype pre-miR-208a hairpin (Fig. 2A). This revealed that the two variants with a high conservation (+42G > T and +68G > T) also alter this hairpin, while the two variants with a low conservation (+5G > A and +41C > T) do not alter the pre-miR-208a hairpin. Variants +42G > T and +68G > T create extra mismatches within the hairpin and thereby enlarge pre-existing loops and reduce thermal stability at the adjacent positions as indicated by an increase in positional entropy (Fig. 2B). Variant +42G > T enlarges an internal bulge at the apical side of the hairpin, thereby effectively reducing the space between the Dicer canonical cleavage site and the internal bulge from 2 to 1 nt at the 3′-arm of the miRNA-duplex. This might affect Dicer processing and shift its cleavage site 1 position towards the basal side of the hairpin [29]. Variant +68G > T enlarges an internal bulge at the basal side of the hairpin two nucleotides 3′ of the canonical Drosha cleavage site. As Drosha’s cleavage is determined by internal bulges in the stem this enlarged internal bulge might affect Drosha processing [7, 30].

### Variants +42G > T and +68G > T impair miRNA maturation

**Figure 3:**
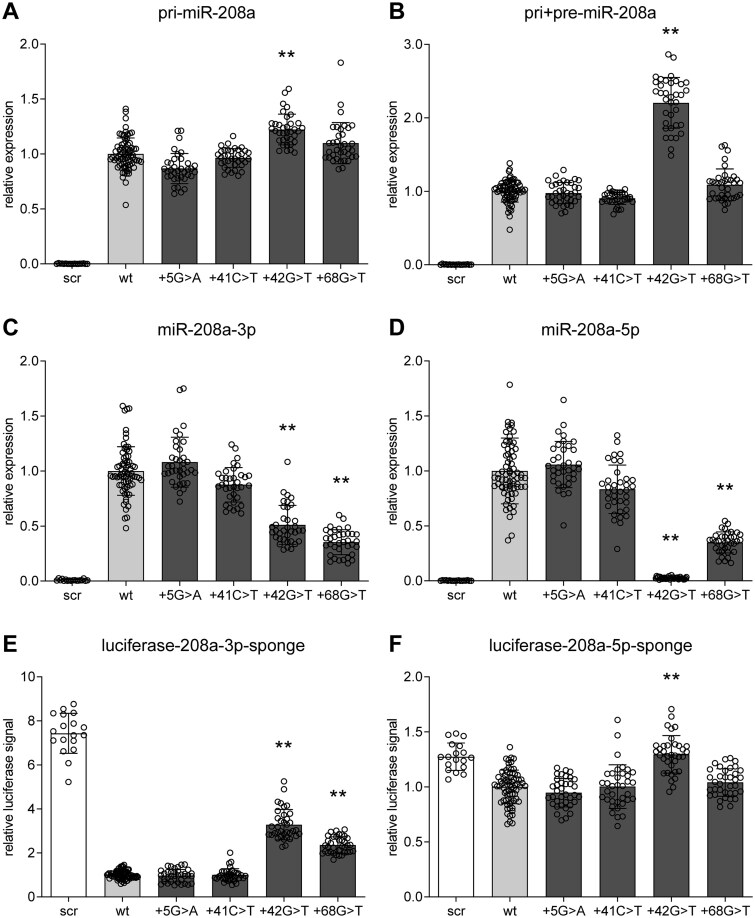
Variants +42G > T and +68G > T impair miRNA formation and function. (A–D) qRT-PCR in COS-7 cells 24 h after overexpression of scrambled control (scr), miR-208a wildtype (wt) or mutant constructs for pri-miR-208a (A), combined pri- and pre-miR-208a (B), mature miR-208a-3p (C) and mature miR-208a-5p (D). All data are normalized to puromycin resistance gene expression and depicted relative to the levels derived from the wildtype construct. (E-F) Luciferase activity in COS-7 cells measured 24 hours after co-transfection of scrambled control (scr), miR-208a wildtype (wt) or mutant constructs with a luciferase-sponge construct, which contains 6 perfect binding sites for miR-208a-3p (E) or miR-208a-5p (F) respectively. Depicted data are normalized to Renilla luciferase activity and relative to wildtype. Data are presented as mean ± SD with a dotplot overlay. Combination of 3 experiments, with triplicates of four different plasmid isolations (one isolation for scrambled) per experiment. ^*^P-value < 0.05 and ^*^^*^P-value < 0.001 compared to wildtype.

To evaluate whether miRNA biogenesis and maturation of miR-208a is, as expected from their secondary structures, indeed affected by the identified variants, we conducted quantitative reverse transcription polymerase chain reaction (qRT-PCR) experiments to determine the levels of miR-208a and its precursors, in miR-208a precursor overexpression experiments containing wildtype miR-208a or miR-208a with the identified variants. We first specifically measured pri-miR-208a, which revealed a significant higher expression (after puromycin-based correction for transfection efficiency, in the +42G > T transfected samples compared to wildtype miR-208a overexpression (fold change [fc] 1.22, *P* < 0.001; Fig. 3A and S2A). +5G > A, +41C > T, and +68G > T did not affect pri-miR-208a expression compared to the wildtype miR-208a overexpression. We also performed a combined measurement of the pri- and pre-miR-208a, as it is impossible to selectively quantitate the pre-miR208a, because its sequence is completely included in pri-miR-208a, which does not allow to design pre-miR-208a specific primers. This combined measurement (pri + pre-miR-208a) again revealed a significant higher expression in the +42G > T miR-208a overexpression compared to the wildtype (fc 2.20, *P* < 0.001; Fig. 3B and S2B). The higher fold-change in the pri + pre-miR-208a compared to the fold change of pri-miR-208a suggests an accumulation at the pre-miR-208a level probably caused by a deficiency in Dicer processing. +5G > A, +41C > T and +68G > T did also not affect pri + pre-miR-208a levels.

To further investigate whether the accumulation of these precursors also resulted in reduced mature miRNA levels we measured both miRNAs derived from the duplex, miR-208a-3p and miR-208a-5p, using a mature miRNA-specific system (TaqMan). This indeed showed that the precursor accumulation due to the +42G > T variant reduced expression of miR-208a-3p (the pre-dominant miRNA derived from this duplex; fc 0.51, *P* < 0.001; Fig. 3C and S2C) and miR-208a-5p was even almost completely absent due to this variant (fc 0.03, *P* < 0.001; Fig. 3D and S2D). Surprisingly, based on the lack of changes in pre- and pri-miR-208a levels, we also detected reduced levels of miR-208a-3p and miR-208a-5p due to the +68G > T variant (for both fc 0.35, *P* < 0.001; Fig. 3C/D and S2C/D). The +5G > A and +41C > T variants did not change expression of the mature miRNA levels compared to the wildtype control.

### Variants +42G > T and + 68G > T impair miR-208a function

To further investigate the functional relevance of the reduced mature miRNA levels due to the +42G > T and + 68G > T variants, we determined the ability of the mature miRNAs derived from the overexpression constructs to inhibit luciferase expression of sponge constructs. These sponge constructs contain six perfectly complementary binding sites behind the luciferase gene (Fig. S3), which activity would be strongly inhibited by binding of the miRNA to the sponge binding sites. The wildtype miR-208a-3p overexpression construct indeed strongly inhibited the luciferase-208a-3p-sponge activity compared to the scrambled negative control (fc 0.13, *P* < 0.001; Fig. 3E and S4A). The variants +42G > T and + 68G > T, which reduced the mature miRNA levels being formed, were also less able to inhibit the luciferase-208a-3p-sponge activity than wildtype miR-208a overexpression. Directly compared to wildtype, this is a significant difference (fc of inhibition levels compared to wildtype miR-208a: +42G > T 3.28, *P* < 0.001; +68G > T 2.36, *P* < 0.001; Fig. 3E and S4A). The variants +5G > A and +41C > T did not alter the luciferase activity compared to the wildtype miR-208a.

Although miR-208a-5p is hardly detected in cardiomyocytes under physiological conditions and miR-208a-3p is the predominant mature miRNA incorporated into the RISC complex [31], we still investigated in our overexpression model whether the reduced miR-208a-5p levels detected due to the +42G > T and +68G > T variants also resulted in less inhibition of a luciferase-miR-208a-5p sponge construct. This experiment revealed that the wildtype miR-208a-5p inhibited the luciferase-208a-5p-sponge activity to a much lesser extent as the wildtype miR-208a-3p miRNA did for its respective sponge (fc 0.78, *P* < 0.001 vs fc 0.13, *P* < 0.001; Fig. 3E/F and S4A/B), which indicated that also in this overexpression model predominantly miR-208a-3p is incorporated in the RISC complex. Nevertheless, the +42G > T variant completely abolished the small inhibitory effect observed from the wildtype miR-208a. The variants +5G > A, +41C > T, and +68G > T did not alter the luciferase activity compared to the wildtype miR-208a.

## Discussion

In this study, we analysed 1640 DCM cases from 3 different cohorts for variants in the cardiac-specific miRNAs, miR-208a and miR-208b. Compared to a general population cohort, gnomAD, we identified 11 enriched variants in the miR-208a locus and no significantly enriched variants in the miR-208b locus. None of these variants were located in the mature miRNA, but 4 were located in pre-miR-208a (+5G > A, +41C > T, +42G > T and +68G > T) and 1 in pre-miR-208b (+2C > T). These pre-miR-208a and pre-miR-208b sequences show low sequence variation between individuals (Fig. 1), similar to previous observations for other miRNAs [10]. Also, across species these sequences are highly conserved (Fig. 1), which suggests that variants in these sequences are not well tolerated and potentially deleterious. Therefore, we hypothesize that the identified pre-miR-208a variants may be associated with the DCM phenotype observed in the carriers. Unfortunately, these carriers were all sporadic DCM cases, i.e. without known affected family members, which prevented segregation analysis with regard to the pathogenicity of the identified variants. Therefore, we used *in silico* prediction and *in vitro* techniques to establish whether the pre-miR-208a variants affect miRNA maturation and function. Interestingly, +42G > T and +68G > T in pre-miR-208a change a highly conserved nucleotide and thereby the secondary pre-miR-208a hairpin structure, which is predicted to affect the miRNA biogenesis by Drosha and Dicer. Indeed, qRT-PCR revealed reduced levels of the mature miR-208a, after overexpression of pre-miR-208a constructs carrying the +42G > T and +68G > T variants. For +42G > T, we also found an accumulation of miRNA-precursors (pri- and pre). Furthermore, these reduced mature miR-208a levels caused by +42G > T and +68G > T, impaired the ability to reduce luciferase activity of miR-208a sponge constructs with 6 perfect binding sites behind the luciferase gene, which indicates also a functional impairment of these variants.

We detected a reduced expression of mature miRNA miR-208a-3p and miR-208a-5p for variants +42G > T and + 68G > T, which might be explained by the increased bulge sizes in the secondary hairpin structure (Figs 2 and 3). For variant +42G > T, we detected both an accumulation of the pri-miR208a and an even stronger accumulation of the pre- and pri-miR208a combined measurement, which suggests a primary effect on Dicer cleavage, as expected from the position of the increased bulge in the secondary hairpin structure. Dicer’s cleavage accuracy is almost perfect when a 2 nt space is present between the cleavage site on the 3′-arm and the apical loop or an internal bulge [29]. Variant +42G > T enlarges the internal bulge and thereby reduces the space between the 3′-arm cleavage site and the internal bulge to 1 nt. Gu et al. [29] demonstrated that such a change promotes non-canonical Dicer cleavage 1 position to the basal side on the 3′-arm (Fig. 2B, grey triangles). Furthermore, internal bulges were also shown to determine Drosha’s cleavage site [7, 30], and therefore variant +42G > T potentially also affects Drosha processing, which could explain why we also detected accumulation of the pri-miR208a. For variant +68G > T, we only detected a reduced level of the mature miRNA and no accumulation of any precursor. Therefore, an effect on Dicer processing seems unlikely, which is supported by the unchanged apical side of the secondary hairpin structure. +68G > T enlarges an internal bulge at the basal side of the hairpin, which might affect Drosha processing. Burke et al. [30] showed that a shift of an internal bulge to the apical side of the hairpin results in non-canonical Drosha cleavage shifted to the apical side. +68G > T enlarges the internal bulge to the basal side, which therefore might result in non-canonical Drosha cleavage to the basal side (Fig. 2B, grey triangles).

Non-canonical cleavage of the hairpin by either Drosha or Dicer can lead to mature miRNAs with slightly changed sequences, so called isomiRs. These non-canonical mature miRNAs are also detected in large sequencing studies in both physiological and pathological circumstances [32]. IsomiRs with a non-canonical 3′-side (3′ isomiRs) are more frequently observed compared to 5′ isomiRs, as these 5′ isomiRs are less well tolerated due to their change in seed sequence (the seed is determined from the 5′-side) and therefore stronger downstream effects [32]. +42G > T and +68G > T could also lead to isomiR formation as they could induce non-canonical cleavage of Dicer and/or Drosha, respectively. Specifically, +42G > T probably shifts Dicer processing to the basal side, leading to a 5′ isomiR of the predominant miR-208a-3p and a 3′ isomiR of miR-208a-5p and +68G > T shifts Drosha processing to the basal side, leading to a 3′ isomiR of the predominant miR-208a-3p and a 5′ isomiR of miR-208a-5p (Fig. 2B, grey triangles). Due to this probable formation of a 5′ isomiR of the predominant miR-208a-3p, the downstream effects of +42G > T are expected to be stronger compared to +68G > T. The TaqMan miRNA system is developed to distinguish between miRNA family members and isomiRs and only detects canonical mature miRNA expression levels. Therefore, isomiR formation could (partially) explain the reduced canonical mature miRNA levels detected in our qRT-PCRs.

Non-canonical cleavage could also affect thermostability of the miRNA duplex and thereby alter which strand of the duplex is build into the RISC complex. Dicer preferentially interacts with the less stable end of the miRNA duplex to subsequently present the strand with the 3′ overhang at this less stable end to Argonaute [33]. This altered strand selection was observed for a variant in mature miR-133a-2-3p (+79C > T) [34], where northern blots after pri-miR-133a-2 overexpression showed a strong preferential strand selection of miR-133a-2-3p from the wildtype precursor and an equal expression of both mature miRNAs from the +79C > T precursor. Our pre-miR-208a variants, located outside the miRNA duplex, could alter duplex themorstability due to the non-canonical cleavage by Drosha or Dicer, meaning that the expression of the predominant pre-miR-208a could shift to miR-208a-5p derived from the 5′-arm of the precursor. However this seems unlikely as we in that case would expect to have detected a stronger reduction in luciferase activity by the constructs with the variants compared to the wildtype miR-208a in our experiments with the miR-208a-5p sponge.

Gain- and loss-of-function studies in mice demonstrated a major role for miR-208a in controlling the ratio between fast twitch αMHC versus slow twitch βMHC and MYH7b (the α/βMHC ratio) and thereby cardiac contractility. In the mouse heart, there is a postnatal shift (around P5) from predominant βMHC expression to predominant αMHC expression accompanied by a similar shift in miR-208b to miR-208a, the miRNAs encoded by *Myh7* and *Myh6* respectively [27]. Cardiac pressure overload due to trans-aortic constriction, results in a strong βMHC upregulation as part of the pathologic hypertrophic response. Interestingly, this hypertrophic response and the βMHC upregulation are completely abolished in miR-208a knockout mice [22]. However, miR-208a knockout mice display a DCM phenotype starting from two months of age, with a progressively declining cardiac function as a result of abnormalities in sarcomere structure [22]. This shows that, reduced miR-208a expression protects against pathological responses in acute cardiac stress, while chronic absence of miR-208a leads to DCM and HF. The latter is likely explained by a downregulation of βMHC and an upregulation of fast skeletal muscle contractile proteins (*Tnni2*, *Tnnt3* and *Mylf*) [22, 27]. This switch to fast and energetically less favourable contractile proteins in miR-208a knockout mice potentially leads to exhaustion of cardiomyocytes and thereby to cardiac dysfunction. Similar to mice, failing human hearts show increased βMHC expression, although in humans βMHC (and thus miR-208b), and not αMHC is the predominant myosin isoform expressed in the ventricles [23, 24]. However, as miR-208a is required for βMHC expression in mice, we similarly expect that in humans ventricular expression of miR-208a is required to maintain ventricular βMHC expression. Therefore, we postulate that loss of miR-208a in humans will also result in a ventricular downregulation of βMHC and an upregulation of fast contractile proteins. Via this mechanism, the two identified loss-of-function variants in miR-208a (+42G > T and +68G > T) may contribute to the DCM phenotype observed in their carriers. Interestingly, we observed less variants in miR-208b compared to miR-208a, both in gnomAD and our DCM cohort (Fig. 1A and B), which suggests that variants in miR-208b are less well tolerated. This could be explained by the fact that miR-208b is the predominant ventricular myomiR, and as a result more important than miR-208a in controlling cardiac contractility.

The hypothesis that miRNA variants contribute to the susceptibility for various cardiovascular diseases has been studied before [35–38]. Zhou et al. [35] analysed a selection of three common (MAF > 0.05) SNPs (rs2910164 in miR-146a-3p, rs11614913 in miR-196a-3p and rs3746444 in miR-499a-3p) in 221 DCM cases and 321 control individuals, which revealed that rs11614913 and rs3746444 were significantly enriched in DCM. However, due to the absence of segregation and/or functional analysis a direct pathophysiological relation between these variants and the DCM phenotype is uncertain. Two studies investigated the association between miRNA variants and hypertrophic cardiomyopathy (HCM) [36, 37]. Palacin et al. [36] found a significant enrichment of variant +85C > A in pri-miR-133a-1 in 250 HCM cases without a sarcomeric mutation compared to 250 control individuals and a significant enrichment of variant -90delA in pri-miR-133b in 120 patients with left ventricular hypertrophy secondary to hypertension. In this cohort, they also identified a miR-208a variant located in the pri-miRNA sequence (-175C > A), which was not further investigated due to absence of relatives for segregation analysis [36]. Curila et al. [37] sequenced miR-1, miR-133a, miR-208a, miR-208b, miR-302a, miR-302c, miR-367 and miR-499 in 56 HCM cases and 60 control individuals. The most frequent identified variant in this cohort rs45489294 in pre-miR-208b (17.8%; 10/56) was not enriched compared to the controls. We also identified this variant (+2C > T in pre-miR-208b) in our DCM cohort without significant enrichment to the gnomAD population. This lack of enrichment in both cohorts suggests that this variant does not contribute to the susceptibility to either HCM or DCM.

Dorn et al. [38] specifically focused on variants in the myomiRs and sequenced 2606 individuals, of which is not reported whether they suffered from a (cardiac) phenotype or not. They only identified 8 variants in the pre-miRNA sequences (1 in pre-miR-208a, 3 in pre-miR-208b and 4 in pre-miR-499) and many more [36] in the pri-miRNA sequences, as we and other groups also observed [10]. They detected the +5G > A variant in pre-miR-208a and the +2C > T variant in pre-miR-208b, which we also identified in our DCM cohort. In addition, they detected two more pre-miR-208b variants, +15 T > C and +21C > T, located in miR-208b-5p. The miR-208a and miR-208b variants, were not further investigated. Three SNPs (rs3746444, rs7267163, and rs140486571) and one novel variant (+49 T > C; in 2 individuals) were identified in pre-miR-499. This +49 T > C variant is located at the 3′-side of mature miR-499-5p and creates an additional internal bulge on the apical side of the pre-miR-499 hairpin. This variant reduced the translational inhibition of canonical miR-499 in luciferase assays with both sponge and endogenous target 3’UTR sequences, possibly due to the formation of an 3′ isomiR (not shown by authors). Furthermore, cardiac-specific overexpression of this mutant miR-499 in mice partially protected against heart failure development compared to wildtype miR-499 overexpression, as a result of a different mRNA targeting profile. Unfortunately, due to the lack of details on the phenotype of the two variant carriers it is unclear whether +49 T > C also associates with cardiac disease in human.

Overall, reports on the contribution of miRNA variants to the susceptibility for cardiomyopathies, and DCM in particular, are rare, which might be explained by miRNA variants being not well tolerated. Here, we show in a relatively large cohort of 1640 cases that variants in miR-208a, although uncommon, can be identified in and associated with DCM. We further show that variants +42G > T and +68G > T cause loss of expression and function of (canonical) miR-208a. Combined with the role of this miRNA in cardiac contractility, this suggests that variants +42G > T and +68G > T in pre-miR-208a may contribute to the DCM phenotype observed in these patients.

## Materials and methods

### Cohorts and sequence analysis

This study was conducted in accordance with the principles of the Declaration of Helsinki. All participants from all centers have given written informed consent and the study was approved by the ethic committees of the participating study centers. The variants identified in this study have been submitted to the Global Variome Shared Leiden Open Variation Database and are accessible through databases.lovd.nl/shared/references/DOI:10.1093/hmg/ddaf069.

#### INHERITANCE project

We used a previously established European cohort of DCM cases, the INHERITANCE (Integrated Heart Research In TrANslational genetics of dilated Cardiomyopathies in Europe) project, for which inclusion criteria, clinical data and DNA collection procedures are described elsewhere [4]. Briefly, between 2009 and 2011 clinical data and DNA from peripheral blood was obtained from patients with either familial or non-familial DCM. Patients with left ventricular dilatation (>117% of the predicted value corrected for age and body surface using the Henry equation) and left ventricular ejection fraction < 45% in the absence of abnormal loading conditions were included. Patients with significant coronary artery disease (stenosis > 50%), a suspicion or evidence for myocarditis or a history of cardio-toxic therapy were excluded.

For the current study, NGS was performed on DNA from 639 DCM cases and first analysed for a variant in the coding region of *MYH6* to exclude these cases for further analysis. The remaining 634 DCM cases were analysed for variants in the miR-208a (hg19 chr14:23857779–23 857 899) and miR-208b (hg19 chr14:23887173–23 887 293) region extended by 1000 nt up- and downstream.

#### 100 000 Genomes project

Data from DCM cases included in the 100 000 Genomes Project (100KGP) was available for the current study through the Genomics England Research Network (Research Network) initiative. Details on the 100KGP and the Research Network are described elsewhere [39]. Briefly, DCM patients were included in 100KGP if there was a clear clinical diagnosis under 40 years of age in the absence of a family history or a diagnosis over 40 years of age and at least one affected relative. DCM patients were not eligible if the diagnosis was unclear, had a history suggestive of a non-genetic cause or if they had a molecular diagnosis for their phenotype (genes required to be tested prior to inclusion: *ABCC9, ACTC1, CSRP3, LMNA, MYH7, PLN, TNNI3, TNNT2, TPM1, TTN, RBM20*). Sequencing, analysis and quality control was performed on a Illumina platform and pipeline.

All cases classified as DCM (504 cases) or DCM and conduction defects (34 cases) from the Cardiovascular Domain of the Rare Disease Programme Release v11 (17/12/2020) were analysed for variants located in intron 29 of *MYH6* (hg19 chr14:23857548–23 858 067) and intron 30 of *MYH7* (hg19 chr14:23886895–23 887 418) containing the miR-208a and miR-208b locus, respectively. Variant extraction was performed on the Genomics England High Performance computer cluster Helix and was cross referenced with clinical data using RlabKey Package in Rstudio. Clinical data was obtained by investigating the presence of Human Phenotype Ontology terms related to DCM (e.g. ‘Dyspnea’) and the specific fields related to family history and age of diagnosis. Further details on the patient carrying the +42G > T variant were obtained through the Patient Explorer tool. All data exports were approved by the Airlock committee.

#### Health in code

For this study, we selected cases who were previously referred to Health in Code for genetic analysis, provided informed consent for the use of their samples for research purposes and had a clinical diagnosis of dilated cardiomyopathy. Cases carrying a pathogenic or likely pathogenic variant in DCM associated genes were excluded. Except for the sex, no clinical or family details were available for the cases. Genomic DNA, isolated from whole blood, was amplified for the miR-208a and miR-208b regions of interest using KAPA polymerase (Roche). Next, we performed Sanger sequencing using BigDye Terminator (Thermo Fisher) to analyse for variants in these regions (hg19 chr14:23857649-23857952 and hg19 chr14:23887006-23887352, respectively). Primer sequences are included in Table S2.

### Genome aggregation database

Version 3.1 from the Genome Aggregation Database (gnomAD) [40] provides whole genome sequencing data from 76 156 unrelated individuals from various disease-specific and population genetic studies and is used as a general population cohort to compare to the DCM cohorts in this study. Variant data was downloaded from https://gnomad.broadinstitute.org/ for miR-208a (GRCh38 chr14:23387491–23 389 691) and miR-208b (GRCh38 chr14:23416891–23 419 091). Variant presence in DCM compared to gnomAD was tested for significance for each DCM cohort separately with an Pearson’s chi-square test using GraphPad Prism 8.4.2. p-values < 0.05 were considered statistically significant. Reported p-values are adjusted for multiple testing (33 tests) using the Bonferroni method. Minor allele frequencies (MAF) were plotted with GraphPad Prism 8.4.2 to generate the gnomAD track in Fig. 1. When multiple variants were detected at the same position the sum of the MAF of these different variants is depicted in this gnomAD track.

### Conservation data

The UCSC genome browser Vertebrate Multiz Alignment & Conservation track was used to generate the sequence alignment panel from Fig. 1 with the following references sequences: human: GRCh37/hg19; chimp: panTro6; mouse: mm10; elephant: loxAfr3; Opossum: momDom5; *xenopus tropicalis*: xenTro9. Nucleotide conservation estimates Genomic Evolutionary Rate Profiling (GERP) and PhyloP, were obtained from the UCSC genome browser (hg19) for the positions of the pre-miR-208a variants identified in DCM, the full pre-miR-208a sequence and intron 29 of *MYH6*. GERP is based on sequences from 35 mammals. For PhyloP the 100 vertebrate and 33 mammal based scores were obtained.

### Hairpin secondary structure

Pre-miRNA secondary hairpin structure predictions were generated by the RNAfold webtool based on the wildtype and mutant miR-208a sequences [28]. The predicted structure is the secondary structure that contributes a minimum of free energy. The colours correspond to the entropy of a certain position. The pre-miR-208a sequence was extended by 10 nucleotides on both the 3′- and 5′-side to illustrate the basal single stranded part of the precursor. The structures were predicted using a constraint for an U-G base pair at the start of the single-stranded basal segment (in Fig. 2 next to the green coloured A-U base pair which is the last base pair of the stem at the basal side).

### Plasmid construction

To construct luciferase-sponge plasmids for miR-208a-3p and miR-208a-5p, complementary oligonucleotides were annealed to form multiple double stranded DNA fragments. Each fragment contained three perfect miRNA binding sites spaced by six nucleotides and flanked by overhangs appropriate for the respective restriction enzyme (Fig. S3). The first fragment for each sponge was cloned into the pMIR-REPORT vector (Ambion) downstream of the luciferase gene, using SpeI and SacI (miR-208a-3p) or SacI and MluI (miR-208a-5p) restriction sites. A second DNA fragment, containing again three perfect miRNA binding sites spaced by six nucleotides and flanked by the appropriate overhangs, was cloned behind the first fragment using SacI and MluI (miR-208a-3p) or MluI and HindIII (miR-208a-5p) restriction sites. This resulted in luciferase-208a-3p-sponge and luciferase-208a-5p-sponge with each a total of six perfect binding sites for the respective miRNA.

The wildtype miR-208a overexpression construct was created by PCR amplification of the pre-miRNA (71 nt) with a flanking sequence at both sides (108 and 167 nt respectively, hg19 chr14:23857638–23 857 983) from human genomic DNA using HOTFIREPol® DNA Polymerase (Solis BioDyne) and ligated into the EcoR1 and BamH1 restriction sites of the pCDH1-MCS1-EF1-Puro vector (System Biosciences). Mutant miR-208a overexpression constructs were created by PCR based mutagenesis of the wildtype construct and cloned into the same restriction sites. The scrambled control miRNA overexpression construct (pCDH1-control-miRNA) is based on the pcDNA™6.2-GW/miR-neg control plasid (Invitrogen), which contains a hairpin structure processed into a mature miRNA, that is predicted not to target any known vertebrate gene. All generated constructs were verified by Sanger sequencing. Primer and oligonucleotide sequences are included in Table S2.

### Cell culturing and transfection

COS-7 cells were cultured in DMEM 41966 (Invitrogen) supplemented with 10% heat-inactivated fetal calf serum and penicillin/streptomycin. For RNA analysis, cells were plated in 12-wells plates at a density of 9^*^10^5^ cells per plate and transfected one day later with 200 ng of wildtype or mutant miR-208a or scrambled overexpression constructs per well using 1.2 μl GeneJammer (Agilent Technologies) according to the manufacturer’s protocol. For luciferase assays, cells were plated in 24-wells plates at a density of 9^*^10^5^ cells per plate and transfected with 5 ng Renilla luciferase plasmid (phRL vector, Promega), 10 ng of either luciferase-208a-3p-sponge, luciferase-208a-5p-sponge or an empty pMIR-REPORT luciferase vector and 100 ng of miRNA overexpression constructs per well using 0.6 μl GeneJammer according to the manufacturer’s protocol.

### RNA isolation and qRT-PCRs

Total RNA was isolated 24 hours after transfection with TRI-Reagent (Sigma) according to the manufacturer’s protocol. 1200 ng of input RNA was DNase (Invitrogen) treated and subsequently divided into 3 equal parts for separate cDNA generation. For the detection of miR-208a precursors and puromycin resistance gene (PuroR), cDNA was generated with the miScript reverse transcription kit (Qiagen) according to the manufacturer’s protocol. For qRT-PCR, 2 μL of this cDNA (eight times diluted) was amplified in a 10 μL reaction containing 2.5 mmol/L MgCl2 and 0.5 μmol/L primers, using LightCycler 480 High Resolution Melting Master (Roche). The following program was applied on a LightCycler 480 system II: 95°C for 10 minutes and 40 cycles of 95°C for 45 seconds, 60°C for 45 seconds, and 72°C for 45 seconds.

To detect mature miR-208a-3p and miR-208a-5p levels, miRNA specific cDNA was generated using the 2 remaining parts of DNase treated RNA using the TaqMan miRNA reverse transcription kit (Applied Biosystems) and the RT-primer of the respective TaqMan miRNA assay (Applied Biosystems; miR-208a-3p Assay ID 000511; miR-208a-5p Assay ID 462036_mat) according to the manufacturer’s protocol. For qRT-PCR, 1.3 μl of fifteen times diluted cDNA was amplified in a 10 μl reaction using 0.5 μl amplification primer of the specific Taqman miRNA assays using LightCycler 480 probes master (Roche). The following program was applied on a LightCycler 480 system II: 95°C for 10 minutes and 50 cycles of 95°C for 15 seconds and 60°C for 1 minute.

MiR-208a precursor and mature miRNA expression levels were analysed using LinRegPCR [41] and corrected for transfection efficiency with PuroR co-expressed from the miRNA overexpression constructs. In most experiments, LinRegPCR could not calculate a N0 value (calculated starting concentration) for miR-208a-5p in the scrambled condition. To include this condition in further analysis and graphical representation missing values were substituted with a very small number, which was calculated using N0 = Nq/(E^(Ct + 8)), where Nq is the threshold derived from LinRegPCR, E is the mean qRT-PCR efficiency of all samples for that amplicon (calculated by LinRegPCR) and Ct is the highest detected Ct-value in the respective experiment. Primer sequences are included in Table S2.

### Luciferase assays

At 24 h after transfection, cells were washed with warm PBS and lysed in 1x lysis buffer (Renilla Luciferase Assay System, Promega). Luciferase and Renilla luciferase (Rluc) activity was measured using a GloMax-Multi Detection System (Promega) according to the manufacturer’s protocol. In white 96-wells plates, 100 μL of luciferin (Biaffin) or 50 μL Rluc substrate (Promega) was added to 20 μL and 10 μL of cell lysate, respectively. Activity was measured with a delay of 3 seconds and an integration time of 1 second. Luciferase measurements were normalized by Rluc activity to control for cell densities and transfection efficiency.

### Statistical analysis *in vitro* data

Luciferase and qRT-PCR experimental results are presented as bargraphs with dot plot overlays and error bars indicate standard deviation. All luciferase and qRT-PCR experiments were performed using triplicates of four different plasmid isolations per overexpression miRNA construct (12 replicates in total per experiment). For scrambled, triplicates of only one plasmid isolation were used. All measurements were repeated in at least three independent experiments with independent statistical analysis (Fig. S2 and S4). The effects of the variants +5G > A, +42G > T and +68G > T were derived from the same experiments, while the effects of variant +41C > T were determined in independent experiments. To compare the effects of the different variants and to present all data in one graph, we combined these experiments by removing the in between-session variation by Factor Correction [42], where both the wildtype and scrambled data points, present in all experiments, where used to calculate the correction factor. In this combined graph, 18 scrambled, 36 miR-208a variant and 72 wildtype data points are depicted (Fig. 3).

Differences between groups were analysed using Kruskal-Wallis test with Dunn’s post hoc test to determine whether the effects of a miR-208a variant significantly differed from the wildtype effects. Analysis were performed using GraphPad Prism 8.4.2 and p-values < 0.05 were considered statistically significant. Reported p-values are adjusted for multiple testing using the Bonferroni method.

## Supplementary Material

Supplementary_material_ddaf069

Supplemental_Table_1_ddaf069

## Notes

*Conflict of interest statement*: Y.M.P. is an inventor on patents, holds minor shares (<5%), and serves as a consultant for biotech and pharmaceutical companies that develop molecules or RNA therapies that target myocardial disease (Forbion Capital, ARMGO BV, Oxitope Pharmaceuticals and Phlox Therapeutics) and received support from Roche Diagnostics. A.J.T. is inventor on patents about RNA therapies that target myocardial diseases. Ph.C. reports personal fees for consultancies, outside the present work, for Amicus, Bristol Myers Squibb, Owkins, Pfizer, Sanofi and Servier.

## References

[1] Pinto YM , Elliott PM, Arbustini E. et al. Proposal for a revised definition of dilated cardiomyopathy, hypokinetic non-dilated cardiomyopathy, and its implications for clinical practice: a position statement of the ESC working group on myocardial and pericardial diseases. Eur Heart J 2016;37:1850–1858. 26792875 10.1093/eurheartj/ehv727

[2] Grünig E , Tasman JA, Kücherer H. et al. Frequency and phenotypes of familial dilated cardiomyopathy. J Am Coll Cardiol 1998;31:186–194. 9426039 10.1016/s0735-1097(97)00434-8

[3] Mahon NG , Murphy RT, MacRae CA. et al. Echocardiographic evaluation in asymptomatic relatives of patients with dilated cardiomyopathy reveals preclinical disease. Ann Intern Med 2005;143:108–115. 16027452 10.7326/0003-4819-143-2-200507190-00009

[4] Haas J , Frese KS, Peil B. et al. Atlas of the clinical genetics of human dilated cardiomyopathy. Eur Heart J 2015;36:1123–1135. 25163546 10.1093/eurheartj/ehu301

[5] Walsh R , Thomson KL, Ware JS. et al. Reassessment of Mendelian gene pathogenicity using 7,855 cardiomyopathy cases and 60,706 reference samples. Genet Med 2017;19:192–203. 27532257 10.1038/gim.2016.90 PMC5116235

[6] Bartel DP . MicroRNAs: genomics, biogenesis, mechanism, and function. Cell 2004;116:281–297. 14744438 10.1016/s0092-8674(04)00045-5

[7] Han J , Lee Y, Yeom KH. et al. Molecular basis for the recognition of primary microRNAs by the Drosha-DGCR8 complex. Cell 2006;125:887–901. 16751099 10.1016/j.cell.2006.03.043

[8] Park JE , Heo I, Tian Y. et al. Dicer recognizes the 5′ end of RNA for efficient and accurate processing. Nature 2011;475:201–205. 21753850 10.1038/nature10198 PMC4693635

[9] MacRae IJ , Zhou K, Li F. et al. Structural basis for double-stranded RNA processing by dicer. Science (1979) 2006;311:195–198. 16410517 10.1126/science.1121638

[10] Saunders MA , Liang H, Li W-H. Human polymorphism at microRNAs and microRNA target sites. Proc Natl Acad Sci 2007;104:3300–3305. 17360642 10.1073/pnas.0611347104 PMC1805605

[11] Mencía A , Modamio-Høybjør S, Redshaw N. et al. Mutations in the seed region of human miR-96 are responsible for nonsyndromic progressive hearing loss. Nat Genet 2009;41:609–613. 19363479 10.1038/ng.355

[12] Hughes AE , Bradley DT, Campbell M. et al. Mutation altering the miR-184 seed region causes familial keratoconus with cataract. Am J Hum Genet 2011;89:628–633. 21996275 10.1016/j.ajhg.2011.09.014 PMC3213395

[13] de Pontual L , Yao E, Callier P. et al. Germline deletion of the miR-17∼92 cluster causes skeletal and growth defects in humans. Nat Genet 2011;43:1026–1030. 21892160 10.1038/ng.915 PMC3184212

[14] Calin GA , Dumitru CD, Shimizu M. et al. Frequent deletions and down-regulation of micro- RNA genes miR15 and miR16 at 13q14 in chronic lymphocytic leukemia. Proc Natl Acad Sci USA 2002;99:15524–15529. 12434020 10.1073/pnas.242606799 PMC137750

[15] Bastami M , Choupani J, Saadatian Z. et al. miRNA polymorphisms and risk of cardio-cerebrovascular diseases: a systematic review and meta-analysis. Int J Mol Sci 2019;20:293. 30642078 10.3390/ijms20020293 PMC6359604

[16] Hogg DR , Harries LW. Human genetic variation and its effect on miRNA biogenesis, activity and function. Biochem Soc Trans 2014;42:1184–1189. 25110023 10.1042/BST20140055

[17] Chen J-F , Murchison EP, Tang R. et al. Targeted deletion of dicer in the heart leads to dilated cardiomyopathy and heart failure. Proc Natl Acad Sci USA 2008;105:2111–2116. 18256189 10.1073/pnas.0710228105 PMC2542870

[18] da Costa Martins PA , Bourajjaj M, Gladka M. et al. Conditional dicer gene deletion in the postnatal myocardium provokes spontaneous cardiac remodeling. Circulation 2008;118:1567–1576. 18809798 10.1161/CIRCULATIONAHA.108.769984

[19] Da Costa Martins PA , De Windt LJ. MicroRNAs in control of cardiac hypertrophy. Cardiovasc Res 2012;93:563–572. 22266752 10.1093/cvr/cvs013

[20] Tijsen AJ , Pinto YM, Creemers EE. Non-cardiomyocyte microRNAs in heart failure. Cardiovasc Res 2012;93:573–582. 22180601 10.1093/cvr/cvr344

[21] Van Rooij E , Quiat D, Johnson BA. et al. A family of microRNAs encoded by myosin genes governs myosin expression and muscle performance. Dev Cell 2009;17:662–673. 19922871 10.1016/j.devcel.2009.10.013 PMC2796371

[22] Van Rooij E , Sutherland LB, Qi X. et al. Control of stress-dependent cardiac growth and gene expression by a microRNA. Science 2007;316:575–579. 17379774 10.1126/science.1139089

[23] Lowes BD , Minobe W, Abraham WT. et al. Changes in gene expression in the intact human heart. Downregulation of alpha-myosin heavy chain in hypertrophied, failing ventricular myocardium. J Clin Invest 1997;100:2315–2324. 9410910 10.1172/JCI119770 PMC508428

[24] Abraham WT , Gilbert EM, Lowes BD. et al. Coordinate changes in myosin heavy chain isoform gene expression are selectively associated with alterations in dilated cardiomyopathy phenotype. Mol Med 2002;8:750–760. 12520092 PMC2039952

[25] Herron TJ , McDonald KS. Small amounts of alpha- myosin heavy chain isoform expression significantly increase power output of rat cardiac myocyte fragments. Circ Res 2002;90:1150–1152. 12065316 10.1161/01.res.0000022879.57270.11

[26] Rundell VLM , Manaves V, Martin AF. et al. Impact of β-myosin heavy chain isoform expression on cross-bridge cycling kinetics. Am J Physiol Heart Circ Physiol 2005;288:H896–H903. 15471982 10.1152/ajpheart.00407.2004

[27] Callis TE , Pandya K, Seok HY. et al. MicroRNA-208a is a regulator of cardiac hypertrophy and conduction in mice. J Clin Invest 2009;119:2772–2786. 19726871 10.1172/JCI36154 PMC2735902

[28] Lorenz R , Bernhart SH, Höner Zu Siederdissen C. et al. ViennaRNA package 2.0. Algorithms Mol Biol 2011;6:26. 22115189 10.1186/1748-7188-6-26 PMC3319429

[29] Gu S , Jin L, Zhang Y. et al. The loop position of shRNAs and pre-miRNAs is critical for the accuracy of dicer processing *in vivo*. Cell 2012;151:900–911. 23141545 10.1016/j.cell.2012.09.042 PMC3499986

[30] Burke JM , Kelenis DP, Kincaid RP. et al. A central role for the primary microRNA stem in guiding the position and efficiency of Drosha processing of a viral pri-miRNA. RNA 2014;20:1068–1077. 24854622 10.1261/rna.044537.114 PMC4114686

[31] Griffiths-Jones S , Grocock RJ, van Dongen S. et al. miRBase: microRNA sequences, targets and gene nomenclature. Nucleic Acids Res 2006;34:D140–D144. 16381832 10.1093/nar/gkj112 PMC1347474

[32] Neilsen CT , Goodall GJ, Bracken CP. IsomiRs--the overlooked repertoire in the dynamic microRNAome. Trends Genet 2012;28:544–549. 22883467 10.1016/j.tig.2012.07.005

[33] Noland CL , Ma E, Doudna JA. SiRNA repositioning for guide strand selection by human dicer complexes. Mol Cell 2011;43:110–121. 21726814 10.1016/j.molcel.2011.05.028 PMC3143821

[34] Ohanian M , Humphreys DT, Anderson E. et al. A heterozygous variant in the human cardiac miR-133 gene, MIR133A2, alters miRNA duplex processing and strand abundance. BMC Genet 2013;14:18. 23497314 10.1186/1471-2156-14-18 PMC3599331

[35] Zhou B , Rao L, Peng Y. et al. Common genetic polymorphisms in pre-microRNAs were associated with increased risk of dilated cardiomyopathy. Clin Chim Acta 2010;411:1287–1290. 20488170 10.1016/j.cca.2010.05.010

[36] Palacín M , Coto E, Reguero JR. et al. DNA variation in myoMIRs of the 1, 133, and 208 families in hypertrophic cardiomyopathy. Cardiogenetics 2011;1:51–54.

[37] Curila K , Tomasov P, Gregor P. Variants in miRNA regulating cardiac growth are not a common cause of hypertrophic cardiomyopathy. Cardiology 2015;130:137–142. 25633875 10.1159/000369247

[38] Dorn GW , Matkovich SJ, Eschenbacher WH. et al. A human 3′ miR-499 mutation alters cardiac mRNA targeting and function. Circ Res 2012;110:958–967. 22374132 10.1161/CIRCRESAHA.111.260752 PMC3320730

[39] The National Genomic Research Library v5.1, Genomics England, One Canada Square, London E14 5AB, United Kingdom. 10.6084/m9.figshare.4530893.v7.

[40] Karczewski KJ , Francioli LC, Tiao G. et al. The mutational constraint spectrum quantified from variation in 141,456 humans. Nature 2020;581:434–443. 32461654 10.1038/s41586-020-2308-7 PMC7334197

[41] Ruijter JM , Pfaffl MW, Zhao S. et al. Evaluation of qPCR curve analysis methods for reliable biomarker discovery: bias, resolution, precision, and implications. Methods 2013;59:32–46. 22975077 10.1016/j.ymeth.2012.08.011

[42] Ruijter JM , Thygesen HH, Schoneveld OJLM. et al. Factor correction as a tool to eliminate between-session variation in replicate experiments: application to molecular biology and retrovirology. Retrovirology 2006;3:2. 10.1186/1742-4690-3-2 PMC1368993 16398936

