# Supplementary material for: Rare DCM associated variants in pre-miR-208a disrupt miRNA maturation and function
Source: Hum Mol Genet. 2025 May 6;34(14):1216–26. doi: 10.1093/hmg/ddaf069 (PMC12228089; doi:10.1093/hmg/ddaf069)
Supplement: Supplementary_material_ddaf069 [file supplementary_material_ddaf069.pdf]

Supplementary material

Rare DCM associated variants in pre-miR-208a disrupt miRNA maturation and function.

Yolan J. Reckman, Jan Haas, Ingeborg van der Made, Simon G. Williams, Iria Gomez Diaz, Mohammed Akhtar, Jens Mogensen, Torsten B. Rasmussen, Eric Villard, Philippe Charron, Perry Elliott, Bernard D. Keavney, Lorenzo Monserrat, Yigal M. Pinto, Benjamin Meder, Anke J. Tijsen.

Contents

Table S1. Identified miR-208a and miR-208b variants in DCM compared to gnomAD v3.1.
Table S2. Oligonucleotides used for sequencing, plasmid construction and qRT-PCR.
Figure S1. pre-miR-208a variant conservation estimates.
Figure S2. Overview of qRT-PCR experiments.
Figure S3. Luciferase-sponge cloning strategy.
Figure S4. Overview of luciferase experiments.

Table S1. Identified miR-208a and miR-208b variants in DCM compared to gnomAD v3.1.

See file: Supplemental Table 1.xlsx

Table S2. Oligonucleotides used for sequencing, plasmid construction and qRT-PCR.

**target**	**description**	**fw/rv**	**sequence (5'-->3')**
luciferase-208a-3p-sponge-1	cloning	fw	ctagtACAAGCTTTTTGCTCGTCTTATctcgagACAAGCTTTTTGCTCGTCTTATctcgagACAAGCTTTTTGCTCGTCTTATgagct
luciferase-208a-3p-sponge-1	cloning	rv	cATAAGACGAGCAAAAAGCTTGTctcgagATAAGACGAGCAAAAAGCTTGTctcgagATAAGACGAGCAAAAAGCTTGTa
luciferase-208a-3p-sponge-2	cloning	fw	cACAAGCTTTTTGCTCGTCTTATctcgagACAAGCTTTTTGCTCGTCTTATctcgagACAAGCTTTTTGCTCGTCTTATa
luciferase-208a-3p-sponge-2	cloning	rv	cgcgtATAAGACGAGCAAAAAGCTTGTctcgagATAAGACGAGCAAAAAGCTTGTctcgagATAAGACGAGCAAAAAGCTTGTgagct
luciferase-208a-5p-sponge-1	cloning	fw	cGTATAACCCGGGCCAAAAGCTCctcgagGTATAACCCGGGCCAAAAGCTCctcgagGTATAACCCGGGCCAAAAGCTCa
luciferase-208a-5p-sponge-1	cloning	rv	cgcgtGAGCTTTTGGCCCGGGTTATACctcgagGAGCTTTTGGCCCGGGTTATACctcgagGAGCTTTTGGCCCGGGTTATACgagct
luciferase-208a-5p-sponge-2	cloning	fw	cgcgtGTATAACCCGGGCCAAAAGCTCctcgagGTATAACCCGGGCCAAAAGCTCctcgagGTATAACCCGGGCCAAAAGCTCa
luciferase-208a-5p-sponge-2	cloning	rv	agcttGAGCTTTTGGCCCGGGTTATACctcgagGAGCTTTTGGCCCGGGTTATACctcgagGAGCTTTTGGCCCGGGTTATACa
miR-208a wildtype	cloning	fw	ACTGgaattcAGACGGAAGTGGAAGAGACAG
miR-208a wildtype	cloning	rv	ACTGggatccTCCTTGAGACACCGTAAGTCC
miR-208a +5G>A	PCR mutagenesis	fw	TGGCCCTGACCCACTTCCTGTGACAGGCGAGCTTTTGGCCCGGG
miR-208a +5G>A	PCR mutagenesis	rv	ACCCGGGCCAAAAGCTCGCCTGTCACAGGAAGTGGGTCAGGGCC
miR-208a +41G>A	PCR mutagenesis	fw	CCCGGGTTATACCTGATGCTCAtGTATAAGACGAGCAAAAAGC
miR-208a +41G>A	PCR mutagenesis	rv	AGCTTTTTGCTCGTCTTATACaTGAGCATCAGGTATAACCCGG
miR-208a +42G>T	PCR mutagenesis	fw	TGGCCCGGGTTATACCTGATGCTCACTTATAAGACGAGCAAAAAG
miR-208a +42G>T	PCR mutagenesis	rv	TTTTTGCTCGTCTTATAAGTGAGCATCAGGTATAACCCGGGCC
miR-208a +68G>T	PCR mutagenesis	fw	AGACGAGCAAAAAGCTTGTTGTTCAGAGGAGCTACCGTCGATC
miR-208a +68G>T	PCR mutagenesis	rv	TGATCGACGGTAGCTCCTCTGAACAACAAGCTTTTTGCTCGTC
pMIR-REPORT	plasmid sequencing	fw	AGAGATCCTCATAAAGGCC
pMIR-REPORT	plasmid sequencing	rv	AGGCGATTAAGTTGGGTA
pCDH1-MCS1-EF1-Puro	plasmid sequencing	fw	AAATGGGCGGTAGGCGTGT
pCDH1-MCS1-EF1-Puro	plasmid sequencing	rv	TCTCTAGGCACCCGTTCAAT
miR-208a-3p	qRT-PCR	fw/rv	Assay ID: 000511 (TaqMan miRNA assay)
miR-208a-5p	qRT-PCR	fw/rv	Assay ID: 462036_mat (TaqMan miRNA assay)
pri-miR-208a	qRT-PCR	fw	ACTCTTTGCTCTGTGAACTCTG
pri-miR-208a	qRT-PCR	rv	CACAGGCTGATCGACGGTAG
pre-miR-208a	qRT-PCR	fw	GAGCTTTTGGCCCGGGTTATAC
pre-miR-208a	qRT-PCR	rv	ACAAGCTTTTTGCTCGTC
puroR	qRT-PCR	fw	CTCGACATCGGCAAGGTGTG
puroR	qRT-PCR	rv	GCCTTCCATCTGTTGCTGCG
miR-208a (Health in Code)	PCR	fw	CATCAGCCCACATGGTGAATA
miR-208a (Health in Code)	PCR	rv	TTTGGAGGCAGTCATGGGTAG
miR-208a (Health in Code)	sequencing	fw	GAGGGCAACAGAAGTGCTTG
miR-208a (Health in Code)	sequencing	rv	CCGTAAGTCCAGCCTAGCAG
miR-208b (Health in Code)	PCR	fw	AATAAGGCTGGCTGTGGACCAA
miR-208b (Health in Code)	PCR	rv	CCATGTCCAGGGTCTGTCTCAG
miR-208b (Health in Code)	sequencing	fw	CCTCAGCAGATCCCACACAG
miR-208b (Health in Code)	sequencing	rv	CTGGAGAAGCCTGAGGACAG

Figure S1

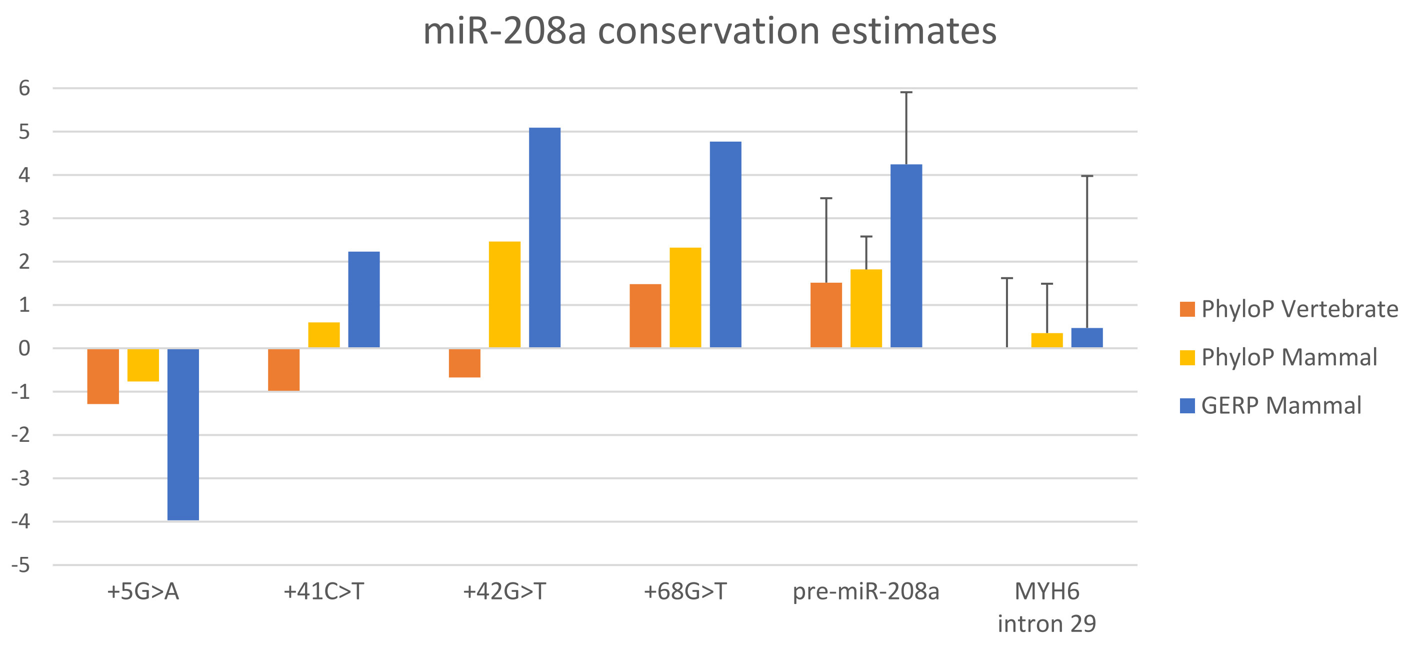

Figure S1. pre-miR-208a variant conservation estimates. Nucleotide conservation estimates were obtained from UCSC genome browser (GRCh37/hg19) for the positions of the pre-miR-208a variants identified in DCM, the full pre-miR-208a sequence (pre-miR) and intron 29 of *MYH6* (intron). GERP is based on sequences from 35 mammals. For PhyloP the 100 vertebrate and 33 mammal based scores were obtained. Pre-miR-208a and *MYH6* intron 29 are presented as mean ± SD.

Figure S2

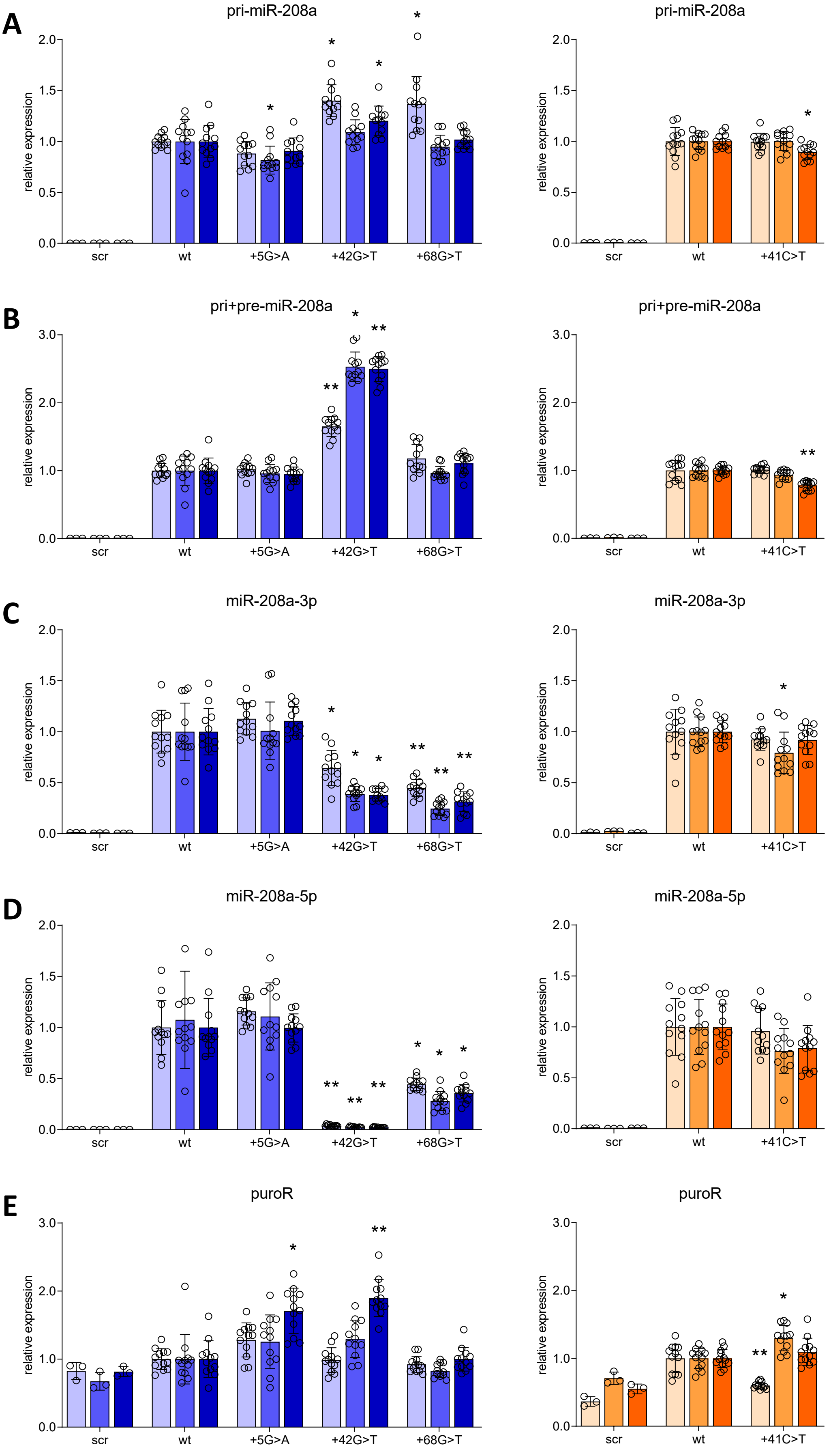

Figure S2. Overview of qRT-PCR experiments. qRT-PCR in COS-7 cells 24 hours after overexpression of scrambled control (scr), miR-208a wildtype (wt) or mutant constructs for pri-miR-208a (A), combined pri- and pre-miR-208a (B), miR-208a-3p (C), miR-208a-5p (D) or puromycin resistance gene (puroR, E). All data are normalized to the expression of the puromycin resistance gene and depicted relative to wildtype. Variants +5G>A, +42G>T and +68G>T were tested in the same experiments, while variant +41C>T was tested independently. Each bar colour denotes a separate experiment (6 experiments in total). Data are presented as mean ± SD with a dotplot overlay. Triplicates of four different plasmid isolations (one isolation for scrambled) per experiment. *p-value <0.05 and **p-value <0.001 compared to wildtype.

Figure S3

Double stranded DNA fragments

```
                                                       miR-208-3p (3′ -> 5′)
                                                      TGTTCGAAAAACGAGCAGAATA
luciferase-208a-3p-sponge-1                           ||||||||||||||||||||||
5′ ctagtACAAGCTTTTTGCTCGTCTTATctcgagACAAGCTTTTTGCTCGTCTTATctcgagACAAGCTTTTTGCTCGTCTTATgagct
3′
3′     aTGTTCGAAAAACGAGCAGAATAgagctcTGTTCGAAAAACGAGCAGAATAgagctcTGTTCGAAAAACGAGCAGAATAc
5′

luciferase-208a-3p-sponge-2
5′     cACAAGCTTTTTGCTCGTCTTATctcgagACAAGCTTTTTGCTCGTCTTATctcgagACAAGCTTTTTGCTCGTCTTATa
3′
3′ tcgagTGTTCGAAAAACGAGCAGAATAgagctcTGTTCGAAAAACGAGCAGAATAgagctcTGTTCGAAAAACGAGCAGAATAtgcgc
5′


                                                       miR-208-5p (3′ -> 5′)
                                                      CATATTGGGCCCGGTTTTCGAG
luciferase-208a-5p-sponge-1                           ||||||||||||||||||||||
5′     cGTATAACCCGGGCCAAAAGCTCctcgagGTATAACCCGGGCCAAAAGCTCctcgagGTATAACCCGGGCCAAAAGCTCa
3′
3′ tcgagCATATTGGGCCCGGTTTTCGAGgagctcCATATTGGGCCCGGTTTTCGAGgagctcCATATTGGGCCCGGTTTTCGAGtgcgc
5′

luciferase-208a-5p-sponge-2
5′ cgcgtGTATAACCCGGGCCAAAAGCTCctcgagGTATAACCCGGGCCAAAAGCTCctcgagGTATAACCCGGGCCAAAAGCTCa
3′
3′     aCATATTGGGCCCGGTTTTCGAGgagctcCATATTGGGCCCGGTTTTCGAGgagctcCATATTGGGCCCGGTTTTCGAGttcga
5′
```

pMIR-REPORT multiple cloning site downstream of the luciferase gene

```
5901 AGGGCGGAAAGTCCAAATTGCTCGAGTGATGAAAGCTGCGCactagtGAG 5950
5951 GgagctcATAGGCCGGCATAGacgcgtGCGTGATATCATAGGTTTAAACA 6000
6001 GTTaagcttAATAAAGGATCTTTTATTTTCATTGGATCTGTGTGTTGGTT 6050
```

Restriction enzymes and spacer (5′ -> 3′)

```
a/ctagt: SpeI
gagct/c: SacI
a/cgcgt: MluI
a/agctt: HindIII
ctcgag:  spacer (Xho1 restriction site)
```

Figure S3. Luciferase-sponge cloning strategy. Complementary oligonucleotides were annealed into multiple double stranded DNA fragments (top). Each fragment contained three perfect miRNA binding sites spaced by six nucleotides and flanked by overhangs appropriate for the respective restriction enzyme. Per sponge, 2 DNA fragments were cloned into the multiple cloning site of pMIR-REPORT downstream of the luciferase gene (middle), resulting in a total of 6 binding sites per sponge. For miR-208a-3p we used the restriction sites SpeI/Sac1 and Sac1/Mlu1 for cloning of the 2 respective fragments in 2 different ligation reactions. And for miR-208-5p we used respectively Sac1/Mlu1 and Mlu1/HindIII. Mature miR-208a-3p and miR-208a-5p sequences are depicted at one bindingsite only to illustrate the respective binding.

Figure S4

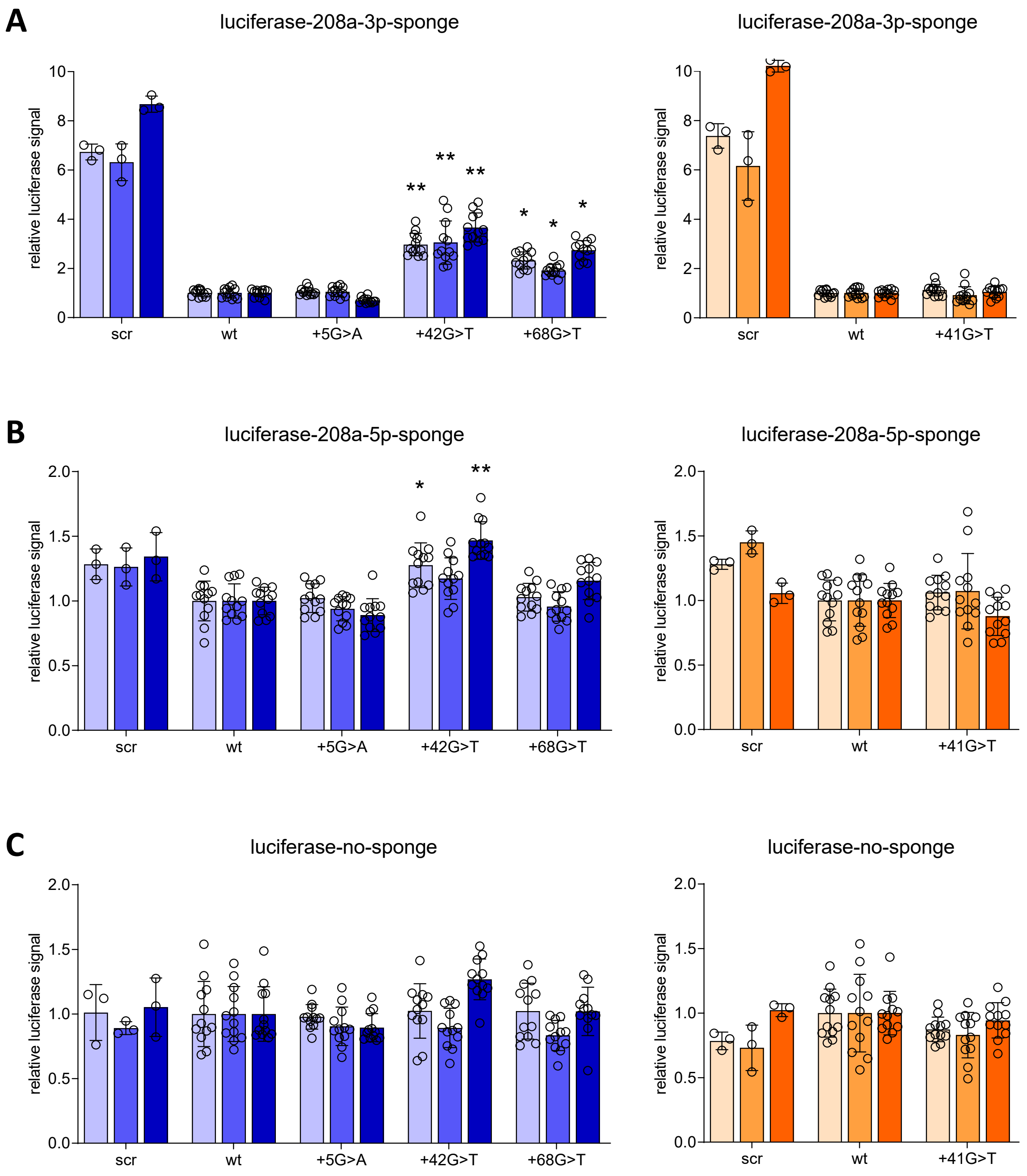

Figure S4. Overview of luciferase experiments. Luciferase activity in COS-7 cells measured 24 hours after co-transfection of scrambled control (scr), miR-208a wildtype (wt) or mutant constructs with a luciferase-sponge construct, which contains 6 perfect binding sites for miR-208a-3p (A), miR-208a-5p (B) or an empty pMIR-REPORT vector without sponge (C). All data are normalized to Renilla luciferase activity and depicted relative to wildtype. Variants +5G>A, +42G>T and +68G>T were tested in the same experiments, while variant +41C>T was tested independently. Each bar colour denotes a separate experiment (6 experiments in total). Data are presented as mean ± SD with a dotplot overlay. Triplicates of four different plasmid isolations (one isolation for scrambled) per experiment. *p-value <0.05 and **p-value <0.001 compared to wildtype.
